# Exploring the influence of AI self-study rooms on K–12 learners’ motivation, self-regulation, enjoyment, and engagement

**DOI:** 10.3389/fpsyg.2026.1768389

**Published:** 2026-03-06

**Authors:** Wei Li, Ya Xu, Le Yao, Yantong Liu

**Affiliations:** 1 Department of Design Media, Zhejiang Fashion Institute of Technology, Ningbo, China; 2 College of Humanities, Sookmyung Women’s University, Seoul, Republic of Korea; 3 School of Humanities, Arts and Education, Shandong Xiehe University, Jinan, China; 4 Department of Computer and Information Engineering, Kunsan National University, Gunsan, Republic of Korea

**Keywords:** AI education, artificial intelligence, engagement, motivation, self-regulated learning

## Abstract

**Background:**

Advances in artificial intelligence (AI) have enabled the development of AI-based self-study rooms that may influence K-12 learners’ motivation, engagement, enjoyment, and self-regulation. This study was guided by Zimmerman’s self-regulated learning framework to explore whether AI-equipped self-study environments can foster more autonomous and effective learning compared to traditional self-study settings.

**Methods:**

A quasi-experimental design was conducted with 383 primary-school students in mainland China, randomly assigned to either an experimental group (AI self-study rooms) or a control group (traditional self-study). Over a 10-week period, participants completed standardized pre- and post-tests, as well as validated scales measuring motivation, engagement, enjoyment, and self-regulation. Data were analyzed using ANCOVA to control for baseline equivalences.

**Results:**

Findings revealed that students exposed to AI self-study rooms recorded notably higher post-intervention scores on all measured constructs than those in traditional self-study settings. ANCOVA results showed that group membership significantly affected motivation (*p* < 0.001, η^2^ = 0.171), self-regulation (*p* < 0.001, η^2^ = 0.238), enjoyment (*p* < 0.001, η^2^ = 0.201), and engagement (*p* < 0.001, η^2^ = 0.220). These outcomes suggest that AI-enhanced environments can better support self-regulated learning processes through personalized feedback, adaptive content recommendations, and data-driven scaffolding.

**Conclusion:**

This study suggests that AI study rooms may be able to provide K-12 students with a more customized, responsive, and engaging learning experience that improves key elements of their learning. Future inquiries could employ longitudinal designs, diversify educational contexts, and integrate broader psychological variables to enrich understanding of how AI-driven tools might shape learners’ trajectories over time.

## 1 Introduction

In recent years, education has undergone substantial transformation driven primarily by rapid technological advancements and their integration into pedagogical practices (Chen et al., 2020; Wang et al., 2023; Huang et al., 2024; Wu et al., 2024). The proliferation of digital technologies—including artificial intelligence (AI), virtual learning environments, and adaptive instructional systems—has enabled educators to refine and personalize teaching methods to accommodate diverse learner needs more effectively (Derakhshan and Yin, 2024; Derakhshan et al., 2024a,b; Zhang et al., 2020; Zhi et al., 2023). Consequently, teaching and learning processes have become more interactive, dynamic, and data-informed, fostering deeper student engagement and supporting improved motivation, enjoyment, and self-regulated learning. Among these innovations, the application of AI within English language education indicates considerable promise for reshaping educational methodologies.

Parallel to these technological advancements, rising demand for supplementary educational resources has led to the emergence of self-study rooms designed to support K-12 students. With China’s “double reduction” policy and the expanding use of AI, AI-equipped self-study rooms have increasingly gained traction, emerging as a notable educational innovation eliciting widespread attention and diverse responses. Beyond instructional enhancements, AI applications also extend into psychological aspects of learning, influencing students’ motivation, engagement, enjoyment, and self-regulation.

Motivation plays a crucial role in education, fundamentally shaping students’ engagement and persistence in learning tasks (Huang et al., 2023; Gao et al., 2024). Yang (2024) provides a comprehensive analysis of studies investigating the impact of AI tools on motivation among EFL learners. Engagement, extending beyond mere participation, encompasses learners’ depth of involvement, interaction, and connectedness with educational activities (Pan et al., 2023). Ebadi and Amini (2024) specifically examined how AI-assisted language learning affects student engagement in EFL contexts. Meanwhile, perceived enjoyment—understood as learners’ anticipated pleasure and expected positive outcomes from technology interactions—significantly influences technology adoption and usage (Collier and Barnes, 2015; Huang and Zou, 2024). Lee et al. (2023) explored how AI-assisted tools enhance EFL learners’ reading enjoyment. Self-regulation, referring to learners’ abilities to manage and adjust their learning behaviors strategically, has also been studied in relation to AI-assisted technologies; for instance, Hsu et al. (2023) investigated the influence of AI-based image recognition tools on students’ self-regulated learning behaviors.

This study adopts Zimmerman’s self-regulated learning (SRL) model due to its comprehensive depiction of cognitive, behavioral, and motivational processes underlying autonomous learning (Zimmerman and Moylan, 2009). Zimmerman’s (2000) SRL model comprises three phases: forethought, performance, and self-reflection. The forethought phase involves task analysis, goal-setting, strategy planning, and motivational beliefs that inform learners’ actions. In the performance phase, learners execute tasks while engaging in self-monitoring and employing strategies to maintain engagement. Lastly, the self-reflection phase entails evaluation of performance outcomes, causal attributions of successes or failures, and resultant self-reactions that guide future learning behaviors.

Despite the widespread implementation of AI in educational settings, empirical research focusing specifically on AI self-study rooms, particularly regarding motivation, engagement, enjoyment, and self-regulation, remains relatively sparse. Addressing this research gap is crucial given the increasing acknowledgment of emotional and psychological factors as integral components of effective learning in AI-enhanced environments. Moreover, AI-driven technologies represent a promising means of cultivating autonomous learning skills and fostering improved self-regulatory capabilities among students (Molenaar, 2022).

Thus, this study investigates the impact of AI self-study rooms on learners’ motivation, engagement, enjoyment, and self-regulation in comparison with traditional self-study contexts.

## 2 Literature review

### 2.1 AI self-study rooms

AI self-study rooms, emerging from rapid advancements in artificial intelligence, initially provided basic automated functions such as tracking learning progress and recommending resources. With developments in machine learning and natural language processing, these platforms now offer advanced features including personalized learning pathways, real-time Q&A interactions, and intelligent assessment tools. Widely adopted in online education and lifelong learning, AI self-study rooms have become essential for enhancing learning efficiency and personalization. Today, these platforms integrate multimodal human-computer interaction, cognitive computing, and adaptive learning technologies to deliver more intelligent and tailored educational experiences.

### 2.2 Cognitive theory

#### 2.2.1 Engagement

Engagement denotes learners’ involvement in diverse activities aimed at attaining educational objectives (Lei et al., 2018). Schaufeli (2013) partially defines engagement as the multifaceted psychological state that underpins active participation in both academic and work-related settings. Ebadi and Amini (2024) investigated the impact of AI-assisted language learning on EFL learners’ language learning engagement by collecting data via motivation, social presence, and human likeness questionnaires, as well as recordings of learners’ interactions with the AI tool. The findings revealed that the AI tool had a significant impact on the learners’ learning motivation and engagement. Xu et al. (2023) analyzed the power of AI tools on the level of 159 Chinese student engagement, indicating a notable rise in classroom engagement following the utilization of AI tools. Almusaed et al. (2023) examined the use of AI to boost learner engagement, with results indicating that AI could transform hybrid education by increasing independence for both students and instructors, thereby creating a more stimulating and interactive learning atmosphere. Huang et al. (2024) emerged as pioneers in substantiating the efficacy of AI in enhancing learner engagement through personalized video recommendations that notably enhanced the success and engagement of moderately motivated learners. Wang and Xue (2024) further examined how AI tools can contribute to increased participation among Chinese EFL students, revealing that the integration of AI tools in EFL classrooms significantly elevated student engagement. As Simpson (2009) succinctly stated, “Engagement at work is critical yet poorly defined; research comprehensively reveals four key areas influencing determinants, consequences, and performance outcomes.” Each of these studies collectively underscores the transformative role of AI in educational settings while also bridging insights from broader engagement research in work environments.

#### 2.2.2 Motivation

AI tools have been shown to exert a notable influence on learners’ motivation. Carpio Cañada et al. (2015) examined how an AI-driven language learning method impacted learners’ motivation and academic performance. Their study revealed that this AI-enhanced approach significantly bolstered learners’ motivation, which subsequently led to improved learning outcomes. Similarly, Ali et al. (2023) investigated the effects of an AI tool, specifically ChatGPT, on the motivation of English language learners and teachers. Their findings indicated that while the AI tool markedly enhanced learners’ writing and reading skills, its impact on speaking and listening skills was relatively more moderate. Ebadi and Amini (2024) explored the influence of AI on the motivation of EFL students, employing questionnaires to measure motivation, social presence, and human-like qualities, as well as observing interactions between students and the AI tool. Their results suggests that the AI tool had a significant positive effect on students’ motivation and engagement. Building on these findings, Alshumaimeri and Alshememry (2023) conducted a systematic review of AI applications in EFL teaching, examining various modern technologies—including augmented reality, virtual reality, and artificial intelligence—and their potential to enhance language skills. Their work also discusses educators’ and students’ perceptions of AI applications and identifies key challenges in implementation. As Alshumaimeri and Alshememry (2023) succinctly summarized: “A systematic review reveals AI’s significant potential in EFL education by enhancing language skills, shaping perceptions, and highlighting implementation challenges.” The research results show that artificial intelligence technology may have a positive impact on improving learning motivation, promoting self-directed learning, and improving learning performance.

#### 2.2.3 Enjoyment

The participants’ perception of their ability to complete tasks independently and their appreciation of the learning content will stimulate their positive learning experiences (Lee et al., 2023). This pleasure refers to the pleasure and positive results obtained from an individual’s interaction with technology (Hong and Tai, 2024). In the realm of artificial intelligence (AI), perceived enjoyment refers to the anticipated satisfaction and pleasure that participants expect when using AI tools (Hong and Tai, 2024). Hong and Tai (2024) studied the relationship between learners’ self-perceptions and learning outcomes in an AI-enhanced gaming activity with 146 participants. Their findings showed that the use of AI was positively correlated with perceived pleasure and negatively correlated with learning anxiety. Jeon (2024) conducted a 16-week course with an artificial intelligence chatbot for 36 learners and found that most participants had a positive attitude towards the chatbot as a learning tool. In a related study, Hernik and Jaworska (2018) posited that enjoyment is a catalyst for effective learning. Gråstén et al. (2012) further explore the relationship between enjoyment and performance in physical education. Collectively, these studies underscore the pivotal role that enjoyment plays in learning, particularly when integrated with emerging digital and AI technologies.

#### 2.2.4 Self-regulation

AI tools not only influence students’ engagement, enjoyment, and motivation but also affect their self-regulation. Wei (2023) examined the differences between AI-assisted and traditional language learning instruction regarding English learning achievement, student motivation, and self-regulated learning among 60 students. In this study, participants were divided into two groups: one received AI-mediated instruction while the other experienced traditional language instruction. Both pre-tests and post-tests were administered to assess achievements in grammar, vocabulary, reading comprehension, and writing skills, and self-report questionnaires measured student motivation and self-regulated learning. The quantitative analysis revealed that the experimental group not only achieved significantly better outcomes in all tested areas but also shows higher motivation and more frequent use of self-regulated learning strategies.

However, Hsu et al. (2023) presented a different perspective by investigating the impact of AI-assisted image recognition technologies on students’ vocabulary knowledge, self-regulation, and anxiety. In their experimental design, the group using AI to display images with related vocabulary showed improvements in vocabulary knowledge and self-regulation, as well as reduced anxiety. Yet, compared to the control group, significant differences emerged only in vocabulary knowledge. These findings suggest that while AI tools generally enhance learning outcomes and motivation, their influence on self-regulation and anxiety may be context-dependent.

### 2.3 Theoretical framework and current research

**Figure 1:**
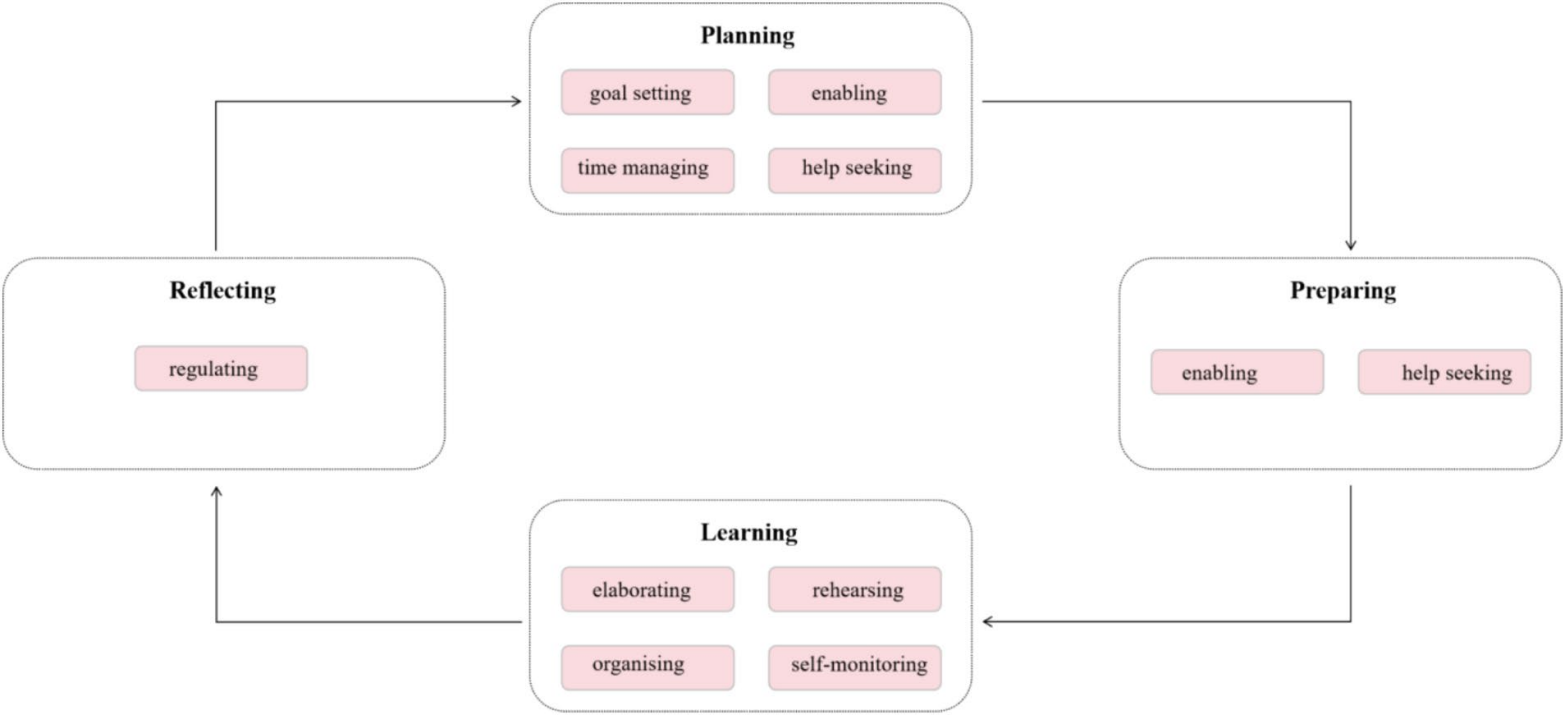
The four stages of the SRL model.

Zimmerman’s self-regulated learning (SRL) model is closely linked to motivation, enjoyment, and engagement, as these three factors play essential roles in each phase of the self-regulation process. Zimmerman’s self-regulation learning model is divided into three phases. The first phase, known as the forethought phase, involves students approaching and analyzing the task, evaluating their ability to complete it successfully, and setting goals and plans for its completion (Panadero and Alonso-Tapia, 2014). If students perceive the learning task as meaningful and personally relevant, their intrinsic motivation increases, leading to more goal-directed behavior. Conversely, extrinsic motivators (e. g., grades or rewards) can also push students to engage in self-study. Performance phase is the second phase. During the performance phase, it is crucial for students to maintain concentration and employ suitable learning strategies (Panadero and Alonso-Tapia, 2014). The process operation of the SRL model is shown in Figure 1.

Engagement is crucial in this stage because it determines the level of effort and persistence students put into their studies. For example, actively participating in learning activities, such as taking notes, summarizing key concepts, or practicing problem-solving. Deeply processing information, making connections between ideas, and using critical thinking skills. In addition, maintaining enthusiasm and interest during the study session, which helps sustain effort and prevent distractions.

The final phase is the self-reflection phase. In this phase, students evaluate their work and identify reasons for their outcomes (Panadero and Alonso-Tapia, 2014). Enjoyment is a key factor in this phase because positive learning experiences reinforce motivation and encourage continued self-regulated learning. Enjoyment reinforces a positive cycle—when students find learning enjoyable, they are more willing to engage in self-study in the future, enhancing long-term academic success.

### 2.4 The current study

**Figure 2:**
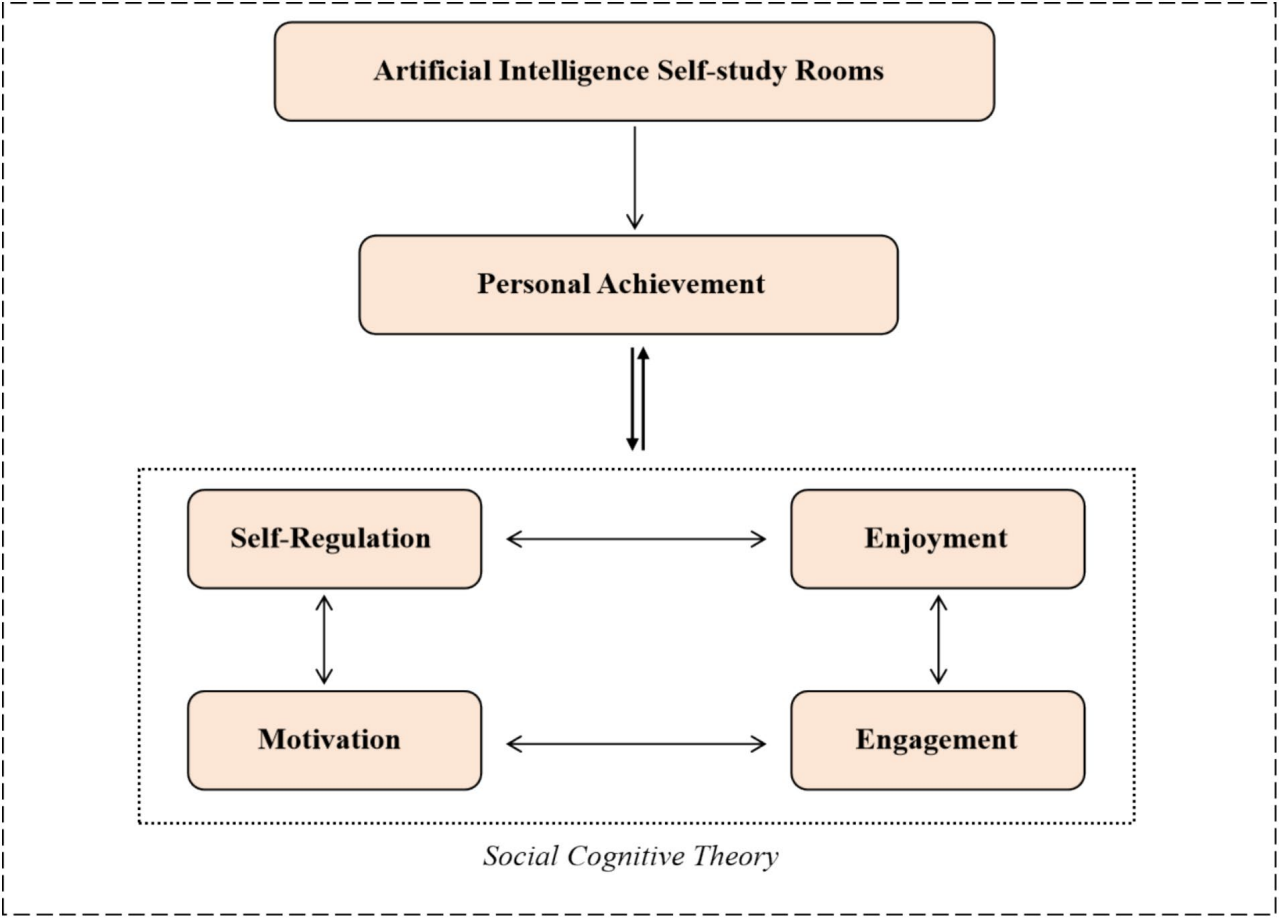
The influence of social cognitive theory on the experimental process.

While existing scholarship has yielded valuable insights into the pedagogical applications of AI tools, three critical limitations emerge when contextualized within AI self-study room environments. First, prior studies predominantly examine isolated learning factors (e.g., engagement or motivation) rather than investigating their synergistic interplay within Zimmerman’s triadic self-regulation framework. Second, the unique affordances of AI self-study rooms—including multimodal interaction protocols, adaptive learning architectures, and real-time metacognitive scaffolding—remain underexplored as holistic mediators of learning experiences. Third, prior researches tends to adopt binary comparisons between AI-enhanced and traditional settings, neglecting the nuanced mechanisms through which integrated AI ecosystems (as opposed to singular AI tools) reconfigure the self-regulated learning cycle across forethought, performance, and reflection phases. The theoretical framework guiding this study, based on social cognitive theory, illustrates the relationship among AI self-study rooms, personal achievement, and key self-regulated learning factors (motivation, engagement, enjoyment, and self-regulation) as depicted in Figure 2.

Acknowledging this gap in the existing literature, this study investigates the influence of AI self-study rooms on learners’ motivation, engagement, enjoyment, and self-regulation compared to traditional learning settings. By simultaneously examining these factors, the study aims to provide a holistic understanding of the benefits and potential challenges associated with AI self-study rooms, thereby informing the broader discourse on educational technology and artificial intelligence in education. The research addresses two key questions:

*Q1*: How do AI self-learning spaces affect learner engagement, enjoyment, motivation and self-regulation compared to traditional self-learning environments?

*Q2*: What significant differences exist in engagement, enjoyment, motivation and self-regulation between AI-enhanced and non-AI-enhanced learning environments?

## 3 Methodology

### 3.1 Participants

This study was conducted in a public primary school in central China. A stratified random sampling method was used to select 383 students in grades K-12 as subjects. Participants were divided into an experimental group (*n* = 197) and a control group (*n* = 186) using a random number table. Both groups took a standardised baseline test to ensure homogeneity of educational background.

The experimental group studied in an AI study room equipped with an intelligent tutoring system, while the control group studied in a traditional study room with the same hardware configuration but without AI technology. All participants and their legal guardians signed an informed consent form, and the experimental protocol was reviewed and approved by the university’s educational ethics committee.

### 3.2 Instruments

#### 3.2.1 Student engagement scale

Given that the participants in this research are K-12 students, this study employed a modified version of the student engagement instrument initially developed by Appleton et al. (2006). The original items were adjusted to ensure clarity and comprehensibility appropriate for younger learners. The resulting scale consists of 12 items measured on a 5-point Likert scale, ranging from “strongly disagree” to “strongly agree.” The reliability coefficient (Cronbach’s alpha) obtained in this study is 0.896.

#### 3.2.2 Enjoyment scale

To accurately measure students’ enjoyment within an AI-driven self-study context, this research adapted the Foreign Language Enjoyment (FLE) scale originally proposed by Dewaele and MacIntyre (2014). Given that AI self-study environments differ markedly from traditional classroom settings—where enjoyment is typically influenced by interactions with teachers, peers, and classroom dynamics—modifications were necessary to address the unique aspects of AI-assisted learning. Specifically, the revised scale incorporated elements reflective of students’ experiences with AI tools, interactive and adaptive feedback mechanisms, and personalized learning pathways. Additionally, the language was adjusted to be age-appropriate and easily understandable for K-12 participants. The modified enjoyment scale consists of 8 items, each rated on a 5-point Likert scale ranging from 1 (strongly disagree) to 5 (strongly agree). The reliability coefficient achieved in this study is 0.844.

#### 3.2.3 Motivation scale

The study utilized an 11-item motivation scale originally developed by Mehdiyev et al. (2017) to assess student motivation at both the start and the end of the experimental procedure. Responses were recorded on a 5-point Likert scale, ranging from “totally agree” to “absolutely disagree.” The overall reliability coefficient calculated in this study is 0.893.

#### 3.2.4 Self-regulation questionnaire (SRQ)

The Self-Regulation Questionnaire (SRQ), as developed by Brown et al. (1999), was employed in this study to measure self-regulatory behavior. In our adaptation, the instrument consists of 16 items, with responses recorded on a 5-point Likert scale ranging from 1 (strongly disagree) to 5 (strongly agree). In a pilot phase, the scale demonstrated strong internal consistency, yielding a reliability coefficient of 0.918.

### 3.3 Data collection procedure

#### 3.3.1 AI system architecture and intervention logic

**Figure 3:**
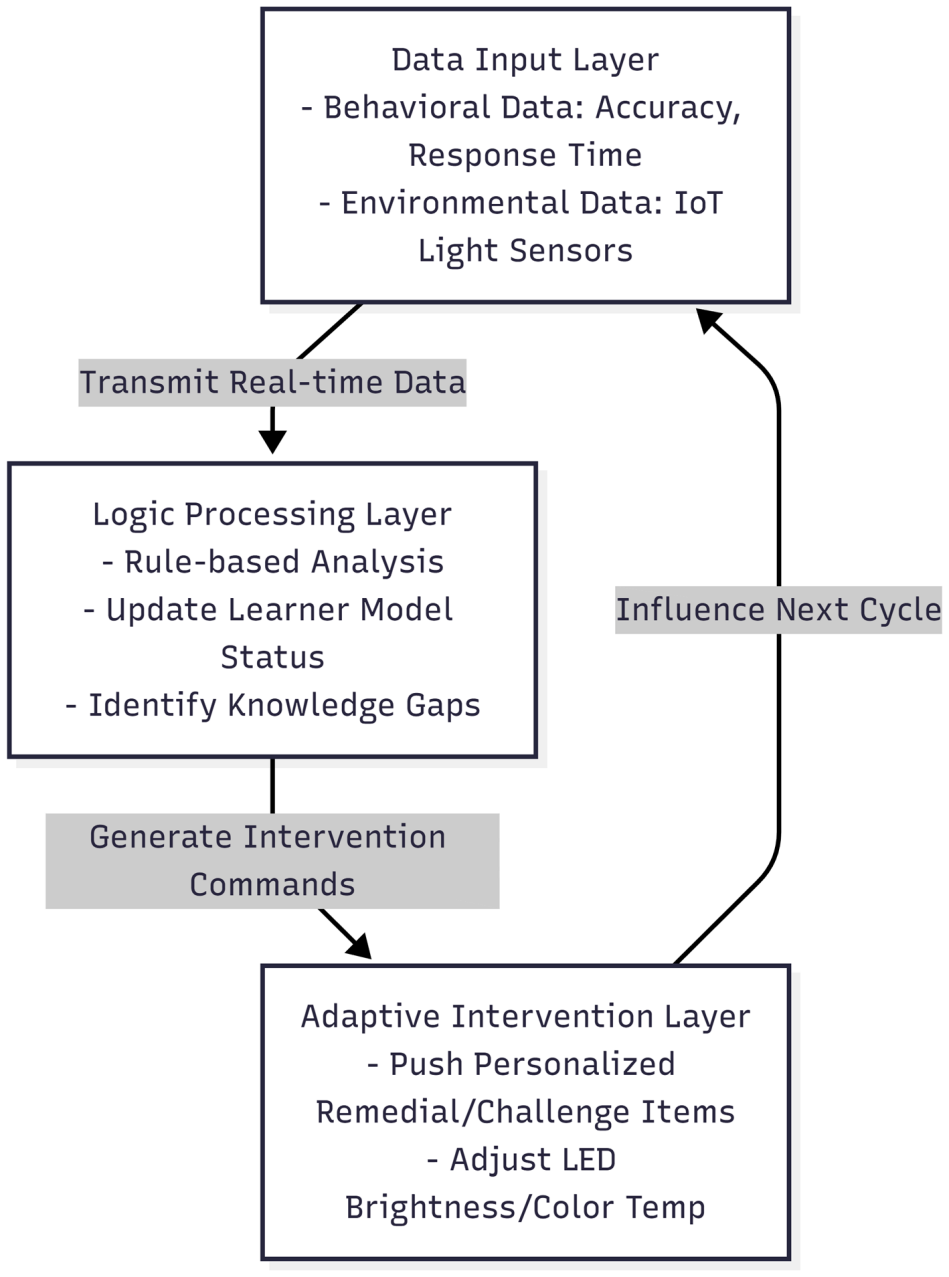
The closed-loop functional architecture and intervention logic of the AI self-study system. Illustrates the system’s operation across three functional layers: the Data Input Layer, which captures real-time behavioral metrics (e.g., accuracy, response time) and environmental sensor data; the Logic Processing Layer, which utilizes a learner model to update mastery status and identify knowledge gaps; and the Adaptive Intervention Layer, which executes personalized content recommendations and adjusts ambient lighting based on the analysis, creating a continuous feedback loop.

To support methodological replicability, the functional architecture of the AI self-study system utilized in this study is detailed below. As illustrated in Figure 3, the platform operates on a closed-loop structure consisting of three functional layers: Data Input, Logic Processing, and Adaptive Intervention.

First, the Data Input Layer continuously records basic behavioral indicators during the learning process, specifically item accuracy, response time, and the use of hints. Simultaneously, simple environmental metrics (e.g., ambient light intensity) are collected through built-in IoT sensors within the study booth. These inputs serve as the foundation for the system’s adaptive decisions.

Second, the Logic Processing Layer analyzes this data using a lightweight learner model organized around topic-level learning objectives. After each task set, the platform updates the student’s estimated mastery status based on recent response patterns and compares them with aggregated historical profiles. This comparison identifies likely knowledge gaps. It is important to note that the decision logic follows predefined adaptive rules configured by the system provider to ensure pedagogical consistency, rather than employing opaque “black-box” deep learning algorithms.

Third, the Adaptive Intervention Layer executes actions based on the updated learner profile. The system recommends individualized practice materials aligned with the student’s current proficiency: remedial items are provided when mastery is insufficient, while more challenging content is unlocked as mastery increases. Additionally, the system controls a programmable LED lighting module, which adjusts brightness or color temperature based on rule-based triggers linked to time-on-task patterns to support visual comfort during extended study periods.

#### 3.3.2 Intervention procedure

The experimental group participated in a 10-week intervention utilizing an AI-supported self-study environment. Participants engaged with AI-driven tools to independently complete academic tasks across subjects including English, Chinese, and mathematics. This environment operated without direct teacher instruction; however, onsite staff were present solely for observational purposes, ensuring students’ adherence to the independent learning structure.

Throughout the intervention, the commercial AI self-study platform automatically logged students’ interactions (for example, time on task and item completion) and used its internal learning analytics to identify learning habits, preferences, and areas of weakness for the purpose of generating adaptive recommendations. In the present study, these log data were used only by the system itself to deliver personalized content. Our statistical analyses focused on standardized pre and post tests and self-report questionnaires, rather than on direct analysis of system log data. Furthermore, AI tools provided targeted feedback on assignments, offered explanations for key concepts, and facilitated interactive learning experiences. Students received assistance in homework completion, and the system recorded their task performance, subsequently generating similar exercises to reinforce learning. Additionally, the AI environment dynamically adjusted the ambient LED lighting according to students’ linguistic input and observed learning states to optimize their comfort and concentration.

At the conclusion of the 10-week period, participants completed questionnaires assessing their levels of engagement, enjoyment, motivation, and self-regulation.

The control group, meanwhile, engaged in a traditional self-study setting, adhering to conventional study room management practices. Over the same 10-week timeframe, a professional instructor supervised student discipline and maintained an appropriate study environment without intervening directly in academic content. Students independently completed predetermined assignments in English, mathematics, and Chinese, followed by the completion of identical post-intervention questionnaires.

### 3.4 Data analysis

Statistical analyses were conducted using SPSS 26.0 software. Descriptive statistics were calculated to summarize pre-test and post-test scores for both the experimental and control groups. Analysis of covariance (ANCOVA) was employed to determine differences between the two groups’ post-test scores, controlling for initial pre-test differences. Given the quasi-experimental nature of this study and the focus on comparing group means to assess intervention effectiveness, ANCOVA was selected as the most robust statistical method to reduce error variance and increase statistical power.

## 4 Results

As mentioned before, the participants of the two groups were asked to fill out four questionnaires before and after the treatment. The descriptive statistics of the results are presented in Table 1. The descriptive statistics indicate that in the experimental group at the pre-test stage, the motivational items ranged from 1.364 to 4.818 with a mean of 3.031 (SD = 0.642) and a median of 3.091, with skewness and kurtosis values of 0.073 and 0.249, respectively. The self-regulation dimension had a mean of 2.965 (SD = 0.597) and a median of 3, with a skewness of 0.104 and kurtosis of 0.128. For enjoyment, the mean was 3.022 (SD = 0.607) with a median of 3, displaying a slight negative skew (−0.341) and low kurtosis (0.118); Engagement had a mean of 2.914 (SD = 0.659), a median of 2.917, with skewness 0.193 and near-zero kurtosis (−0.013). At the post-test stage, the experimental group showed improvement across all indicators: the mean for Motivation increased to 3.756 (SD = 0.806), Self-regulation to 3.831 (SD = 0.708), Enjoyment to 3.833 (SD = 0.746), and Engagement to 3.853 (SD = 0.704), with medians shifting to 4 or near 4, indicating a concentration of higher scores.

**Table 1:** Descriptive statistics of the scores obtained by two groups.

Group	Time	Test	Min	Max	Mean	SD	Skewness	Kurtosis	*N*
Experimental	Pretest	Motivation	1.364	4.818	3.031	0.642	0.073	0.249	197
		Self-regulation	1.375	4.813	2.965	0.597	0.104	0.128	197
		Enjoyment	1	4.375	3.022	0.607	−0.341	0.118	197
		Engagement	1.333	4.917	2.914	0.659	0.193	−0.013	197
	Posttest	Motivation	1.636	4.818	3.756	0.806	−1.035	−0.004	197
		Self-regulation	1.188	4.875	3.831	0.708	−1.175	0.755	197
		Enjoyment	1.25	4.75	3.833	0.746	−1.219	0.834	197
		Engagement	1.333	4.917	3.853	0.704	−1.101	0.854	197
Control	Pretest	Motivation	1.364	4.273	2.949	0.73	−0.935	−0.509	186
		Self-regulation	1.313	3.875	2.912	0.721	−1.007	−0.403	186
		Enjoyment	1.125	4	2.952	0.748	−0.896	−0.402	186
		Engagement	1.333	3.833	2.922	0.705	−0.996	−0.444	186
	Posttest	Motivation	1.364	4.636	3.118	0.566	−0.707	0.414	186
		Self-regulation	1.125	4.813	3.133	0.516	−0.908	1.706	186
		Enjoyment	1.375	5	3.164	0.576	−0.701	0.995	186
		Engagement	1.25	4.75	3.184	0.537	−0.715	1.572	186

All variables were measured on a 5-point Likert scale (1 = strongly disagree, 5 = strongly agree). Skewness and kurtosis values indicate approximate normality for both groups. *N* = sample size for each condition.

In the control group, the pre-test mean scores for motivation, Self-regulation, enjoyment, and engagement were slightly lower at 2.949 (SD = 0.73), 2.912 (SD = 0.721), 2.952 (SD = 0.748), and 2.922 (SD = 0.705), respectively. At post-test, these means increased to 3.118 (SD = 0.566), 3.133 (SD = 0.516), 3.164 (SD = 0.576), and 3.184 (SD = 0.537), although the improvement was less pronounced compared to the experimental group. Overall, the experimental group demonstrated improvements following the intervention, and the distribution characteristics (skewness and kurtosis) of the variables indicate approximate normality data (see Tables 2–6).

**Table 2:** *T*-test results of the four groups.

Source	Group (mean values ± standard deviation)		*t*	*p*
	Experiment (*n* = 197)	Control (*n* = 184)		
Motivation	3.031 ± 0.642	2.949 ± 0.730	1.166	0.245
Self-regulation	2.965 ± 0.597	2.912 ± 0.721	0.780	0.436
Enjoyment	3.022 ± 0.607	2.952 ± 0.748	0.996	0.320
Engagement	2.914 ± 0.659	2.922 ± 0.705	−0.114	0.909

*p < 0.05, **p < 0.01, ***p < 0.001.

**Table 3:** ANCOVA on motivation scores.

Source	Type III sum of squares	df	Mean square	*F*	p	Partial eta squared
Intercept	150.345	1	150.345	306.954	0.000***	0.448
Group	38.300	1	38.300	78.196	0.000***	0.171
Pretest	0.849	1	0.849	1.734	0.189	0.005
Residual	185.143	378	0.490			

Remark: *R*^2^ = 0.242; *p < 0.05, ** *p* < 0.01, ***p < 0.001.

**Table 4:** ANCOVA on self-regulation scores.

Source	Type III sum of squares	*df*	Mean square	*F*	*p*	Partial eta squared
Intercept	158.332	1	158.332	408.397	0.000***	0.519
Group	45.829	1	45.829	118.210	0.000***	0.238
Pretest	0.592	1	0.592	1.528	0.217	0.004
Residual	146.548	378	0.388			

Remark: *R*^2^ = 0.242; *p < 0.05, **p < 0.01, ***p < 0.001.

**Table 5:** ANCOVA on enjoyment scores.

Source	Type III sum of squares	df	Mean square	*F*	*p*	Partial eta squared
Intercept	181.158	1	181.158	403.663	0.000***	0.516
Group	42.595	1	42.595	94.912	0.000***	0.201
Pretest	0.004	1	0.004	0.008	0.928	0.000
Residual	169.641	378	0.449			

Remark: *R*^2^ = 0.201; *p < 0.05 **, *p* < 0.01 ***, *p* < 0.001.

**Table 6:** ANCOVA on engagement scores.

Source	Type III sum of squares	df	Mean square	*F*	*p*	Partial eta squared
Intercept	165.762	1	165.762	418.699	0.000***	0.526
Group	42.237	1	42.237	106.686	0.000***	0.220
Pretest	0.378	1	0.378	0.954	0.329	0.003
Residual	149.650	378	0.396			

*R*^2^ = 0.223; *p < 0.05, **p < 0.01, ***p < 0.001.

The baseline *t*-test results indicate that there were no significant differences between the experimental group (*n* = 197) and the control group (*n* = 184) on the measured variables prior to the intervention. Specifically, the mean Motivation score was 3.031 (SD = 0.642) for the experimental group and 2.949 (SD = 0.730) for the control group, with *t* = 1.166 and *p* = 0.245. For Self-Regulation, the mean scores were 2.965 (SD = 0.597) and 2.912 (SD = 0.721), respectively (*t* = 0.780, *p* = 0.436). The Enjoyment dimension had mean scores of 3.022 (SD = 0.607) and 2.952 (SD = 0.748) for the experimental and control groups, respectively (*t* = 0.996, *p* = 0.320). Finally, engagement scores were 2.914 (SD = 0.659) and 2.922 (SD = 0.705), with *t* = −0.114 and *p* = 0.909. Since all *p*-values exceed the conventional significance level of 0.05, the two groups were statistically equivalent at baseline, providing a robust foundation for subsequent comparisons in the study.

To answer the research question of the study, four sets of Analysis of Covariance (ANCOVA) were run. In this covariance analysis with Motivation Items as the dependent variable, group membership was examined as a predictor while controlling for the pre-test total score. The intercept was highly significant (*F* = 306.954, *p* < 0.001, Partial η^2^ = 0.448), indicating a strong overall model fit. Crucially, the group factor showed a significant effect on Motivation Items (*F* = 78.196, *p* < 0.001, Partial η^2^ = 0.171), which demonstrates that there is a significant difference between the experimental and control groups in terms of motivation, accounting for approximately 17.1% of the variance. The pre-test total score did not reach significance (*F* = 1.734, *p* = 0.189, Partial η^2^ = 0.005), suggesting that baseline motivation did not substantially influence the outcome. The overall model explained 17.6% of the variance (*R*^2^ = 0.176).

For the Self-regulation dimension, a covariance analysis was conducted controlling for the pre-test total score to assess the impact of group membership. The intercept was significant (*F* = 408.397, *p* < 0.001, Partial η^2^ = 0.519), indicating a strong baseline model fit. Additionally, the group effect was highly significant (*F* = 118.210, *p* < 0.001, Partial η^2^ = 0.238), demonstrating a significant difference between the experimental and control groups in self-management, with the group factor accounting for 23.8% of the variance. The pre-test total score did not have a significant effect (*F* = 1.528, *p* = 0.217, Partial η^2^ = 0.004). Overall, the model explained 24.2% of the variance (*R*^2^ = 0.242) in Self-regulation.

In the covariance analysis for the Enjoyment dimension, the pre-test total score was controlled to assess the impact of group membership on Enjoyment scores. The intercept was highly significant (*F* = 403.663, *p* < 0.001, Partial η^2^ = 0.516), indicating a strong baseline model. The group factor also had a significant effect (*F* = 94.912, *p* < 0.001, Partial η^2^ = 0.201), suggesting that there is a significant difference in Enjoyment between the experimental and control groups, with group membership accounting for 20.1% of the variance. The pre-test total score had virtually no impact on Enjoyment (*F* = 0.008, *p* = 0.928, Partial η^2^ = 0.000). Overall, the model explained 20.1% of the variance in Enjoyment (*R*^2^ = 0.201).

For the Engagement dimension, a covariance analysis was conducted controlling for the pre-test total score to evaluate the effect of group membership on Engagement scores. The intercept was significant (*F* = 418.699, *p* < 0.001, Partial η^2^ = 0.526), indicating a strong baseline model. The group factor also had a significant impact on Engagement (*F* = 106.686, *p* < 0.001, Partial η^2^ = 0.220), demonstrating a significant difference between the experimental and control groups, with group membership accounting for 22.0% of the variance. The pre-test total score did not have a significant effect (*F* = 0.954, *p* = 0.329, Partial η^2^ = 0.003). Overall, the model explained 22.3% of the variance in Engagement (*R*^2^ = 0.223), highlighting the important role of group differences in influencing Engagement scores.

**Figure 4:**
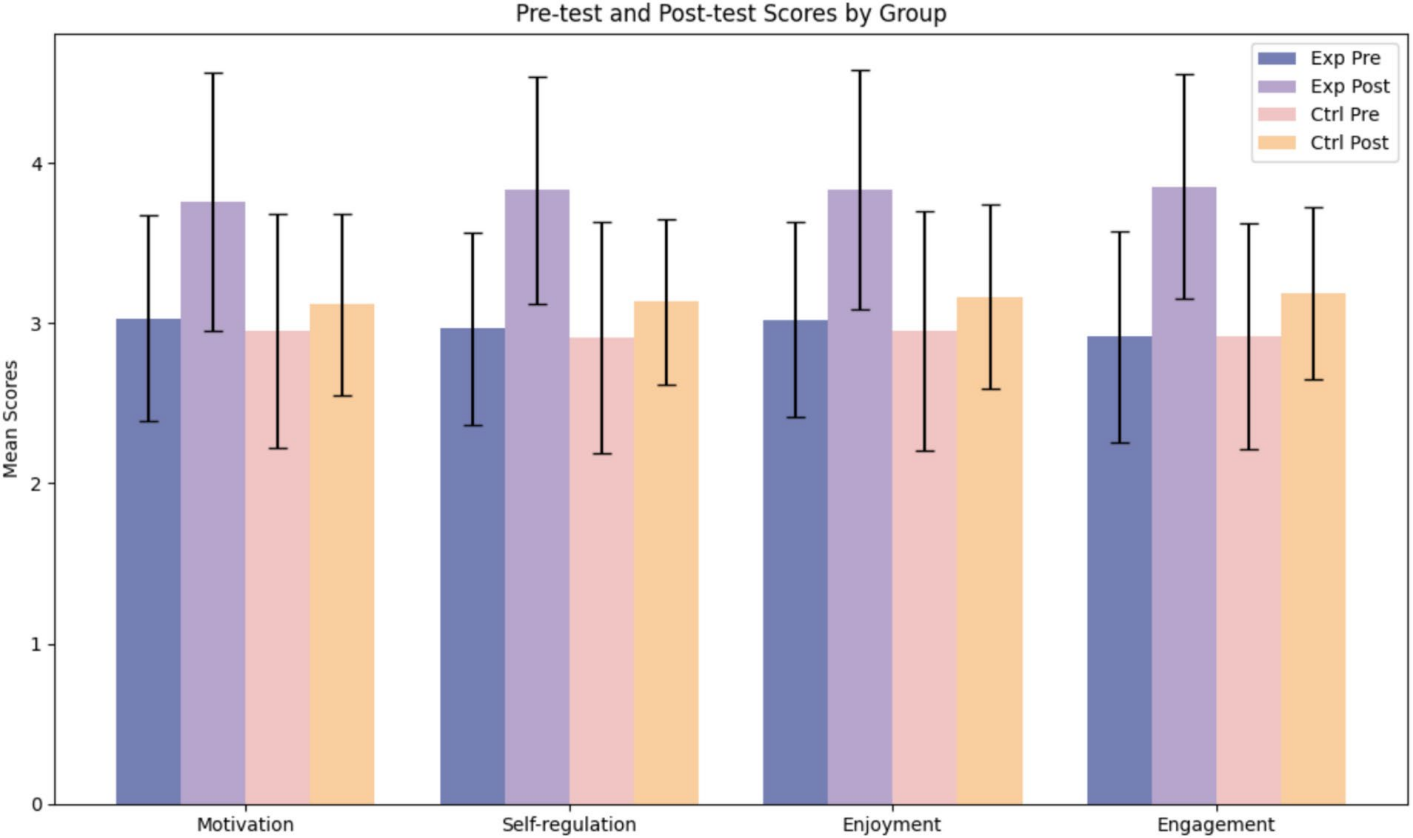
Pre-test and post-test scores by group.

Figure 4 illustrates the mean scores of the experimental and control groups for the four dimensions (Motivation, Self-regulation, Enjoyment, and Engagement) at both the pre-test and post-test stages. The grouped bar chart clearly demonstrates the significant improvements in the experimental group compared to the control group after the intervention, as evidenced by the higher post-test scores. Additionally, error bars representing the standard deviations are included to indicate the variability of the data.

## 5 Discussion

The findings of this quasi-experimental study indicate that participation in AI self-study rooms was associated with higher levels of engagement, enjoyment, motivation, and self-regulation in English learning compared with traditional self-study rooms. These outcomes are well framed by the self-regulated learning model, which outlines how AI environments can support the cyclical processes of planning, performance, and reflection (Zimmerman and Moylan, 2009).

Students in the AI self-study rooms reported higher post-intervention motivation. One plausible explanation is that these environments allow learners to experiment, make mistakes, and take risks without fear of negative evaluation, which may in turn support more active participation. This non-judgmental setting encourages risk-taking and active learning, supporting findings from Wei (2023) that emphasize the positive impact of immediate, personalized feedback on learner motivation.

Similarly, the study found that students experienced higher levels of enjoyment in the AI self-study rooms. The immersive features and personalized learning pathways provided by these environments not only improve learning efficiency but also foster a sense of autonomy and achievement. This finding is consistent with research by Wang and Xue (2024), which highlights the role of interactive digital tools in enhancing learner enjoyment and engagement.

The research further indicates that students assigned to AI self-study rooms reported greater engagement. The adaptive features of AI tools may enable learners to manage their study time more effectively and interact with content in a more personalized way, which is consistent with Xu et al. (2023), who reported increased engagement levels among students using AI-assisted learning tools.

Another significant outcome is the improvement in students’ self-regulated learning abilities. With the support of continuous, adaptive feedback and instructional scaffolding, learners became better at setting goals, monitoring progress, and reflecting on their learning experiences (Xu et al., 2022). This enhancement of self-regulation is critical for long-term academic success and underscores the transformative potential of AI in education.

To be more specific, the functional affordances of the AI self-study room align closely with the three cyclical phases of Zimmerman’s (2000) SRL model. First, in the Forethought Phase, the system’s adaptive recommendation engine acts as a scaffolding tool for task analysis and goal setting. By automatically identifying knowledge gaps and suggesting a tailored learning sequence, the AI reduces the cognitive load associated with planning, allowing students to approach tasks with clearer direction and higher self-efficacy. Second, during the Performance Phase, features such as real-time feedback and the environmental lighting control system support students’ self-control and self-observation. The immediate hints provided during problem-solving help maintain attention and prevent frustration-induced disengagement, while the adaptive lighting minimizes physical fatigue, thereby sustaining behavioral engagement. Third, in the Self-Reflection Phase, the system provides data-driven summaries of mastery levels and generates targeted remedial exercises. This facilitates accurate self-judgment and causal attribution, enabling learners to objectively evaluate their progress and strategically adjust their efforts for subsequent learning cycles, rather than attributing failure to lack of ability.

Finally, when comparing the experimental group with the control group, the experimental group shows superior motivational outcomes. These findings are in line with studies by Carpio Cañada et al. (2015) and Ebadi and Amini (2024), suggesting that AI tools not only promote self-regulation but also contribute to higher levels of both intrinsic and extrinsic motivation. Additionally, Jeon (2024) noted that AI-mediated environments can leverage timely feedback to sustain and boost learners’ motivation over time.

From a psychological perspective, this study underscores the role of environmental and technological factors in shaping learner motivation, engagement, and self-regulation. Future research could explore individual psychological differences, such as personality traits or anxiety levels, to better understand how these variables interact with AI-based learning environments.

When students study in the AI self-study rooms, self-control ability and self-regulation ability are crucial. The Self-regulation Learning Model can provide effective theoretical support for students’ self-study and self-management abilities.

In addition, AI can increase students’ confidence and motivation to learn by providing timely feedback, adjusting task difficulty, and providing guided scaffolding to increase self-efficacy. AI tools offer many advantages, such as the delivery of tailored content, immediate feedback and the ability to adapt to individualised learning pathways.

## 6 Conclusion

This study provides empirical evidence that AI self-study rooms significantly enhance K-12 students’ motivation, engagement, enjoyment, and self-regulation in English learning. Grounded in Zimmerman’s (2000) self-regulated learning model, the findings suggest that intelligent systems have the potential to effectively support the cyclical processes of learning—offering adaptive goal-setting during the forethought phase, real-time monitoring in the performance phase, and data-driven evaluation during reflection.

The study highlights that AI self-study rooms, with their personalized learning pathways, interactive feedback, and autonomous scaffolding, offer distinct advantages over traditional self-study methods. The results suggest that these environments not only facilitate immediate improvements in learning outcomes but also have the potential to foster sustained academic growth. Importantly, the study underscores that the integration of AI should complement rather than replace teacher guidance, thereby promoting a balanced human-AI collaboration.

Methodologically, this research establishes a replicable framework for assessing multi-dimensional learning outcomes within intelligent tutoring systems. Future research should explore longitudinal effects to verify the durability of observed improvements, conduct comparative analyses across different demographic groups and educational contexts, and investigate the specific contributions of various AI features to learning outcomes.

Despite the valuable insights provided by this study, it is important to acknowledge its limitations. The study was conducted in a controlled educational setting, focusing on a specific group of primary school students from a single school.

First, although the AI self-study environment implements internal learning analytics and adaptive algorithms based on detailed system log data, the present study relied on aggregate pre and post measures obtained from standardized tests and self-report scales. We did not conduct a process-level analysis of clickstream or log data. Future research should incorporate learning analytics methods to trace how specific adaptive features, feedback patterns, and interaction trajectories contribute to changes in motivation, engagement, enjoyment, and self-regulation.

Therefore, caution should be exercised when generalizing the findings to broader educational contexts and more diverse student populations. Future research should aim to address these limitations by using larger and more representative samples. Additionally, while this study relied on established scales with high internal consistency (Cronbach’s alpha), future studies could use advanced psychometric evaluations, such as confirmatory factor analysis (CFA), to validate the structural properties of modified instruments in AI-based learning contexts even further.

Moreover, the scope of this study was largely confined to the short-term impacts of AI-assisted instruction, due to the limited duration of the research. To gain a more comprehensive and holistic understanding of the phenomenon, it is essential to conduct long-term follow-up studies. These extended studies will help to assess the sustainability of the observed improvements in achievement, motivation, and self-regulated learning.

Finally, future research could benefit from integrating psychological constructs such as learner anxiety, self-efficacy, or cognitive load, to deepen the understanding of how influence the effectiveness of AI-enhanced learning environments. Subsequent studies will also utilize advanced analytical techniques, such as Structural Equation Modeling (SEM) and mediation analysis, to unravel the complex structural relationships and internal mechanisms between AI usage, learner psychology, and academic outcomes.

## Data Availability

The raw data supporting the conclusions of this article will be made available by the authors, without undue reservation.
